# Supplementary material for: Patterns of Equine Small Strongyle Species Infection after Ivermectin Intervention in Thailand: Egg Reappearance Period and Nemabiome Metabarcoding Approach
Source: Animals (Basel). 2024 Feb 8;14(4):574. doi: 10.3390/ani14040574 (PMC10886017; doi:10.3390/ani14040574)
Supplement: Supplementary file 1 [file animals-14-00574-s001.zip › animals-2821716-supplemetary text.pdf]

Analysis of FECRT data from

fecrt.com data analysis tool

2023-09-15

Efficacy classification

Efficacy classification: Susceptible

[Based on an expected efficacy of 99.9% and a lower efficacy threshold of 96%]

Detailed results

The statistical method automatically chosen for your dataset was the BNB method (version B): see further details of statistical methods below the summary statistics

Summary statistics

Number of animals: 11

Mean of pre-treatment data: 981.82

Mean of post-treatment data: 4.55

Variance of pre-treatment data: 463136.36

Variance of post-treatment data: 227.27

Estimated over-dispersion (k) of pre-treatment data: 2.92

Estimated over-dispersion (k) of post-treatment data: 20

Estimated within-animal correlation: -0.23

Results from the Delta method (Levecke et al.)

Classification: Susceptible

90% CI = 98.5% - 100%

Notes:

- This method is non-parametric, so is robust to distributional assumptions, and variances of the pre-treatment and post-treatment data are estimated independently
- This method cannot be used when the post-treatment data are all zero
- This method may give misleading results with fewer than five observations, and when fewer than three post-treatment observations are non-zero, due to unstable variance estimates
- This is the preferred method when the sample size is greater than or equal to 5, and where at least three post-treatment observations are non-zero

Results from the WAAVP method (Coles et al. and Pepper et al.)

Classification: Susceptible

90% CI = 96.8% - 99.9%

Notes:

- This method is non-parametric, so is robust to distributional assumptions, and variances of the pre-treatment and post-treatment data are estimated independently
- This method cannot be used when the post-treatment data are all zero
- This method may give misleading results with fewer than five observations, and when fewer than three post-treatment observations are non-zero, due to unstable variance estimates

Results from the BNB method (Denwood et al.) version A

Classification: Susceptible

Test for Resistance: p = 0.199; Test for Susceptibility: p = 0.004

Notes:

- This method is parametric, and assumes that the data follow a negative binomial distribution: the classification will be unavailable if the multiplication factor you entered does not match the data
- The over-dispersion is estimated independently for the pre-treatment and post-treatmentdata
- This method may give misleading results with fewer than five observations, and when fewer than three post-treatment observations are non-zero, due to unstable estimates of over-dispersion

Results from the BNB method (Denwood et al.) version B

Classification: Susceptible

Test for Resistance: p = 0.197; Test for Susceptibility: p = 0.011

Notes:

- This method is parametric, and assumes that the data follow a negative binomial distribution: the classification will be unavailable if the multiplication factor you entered does not match the data
- The over-dispersion is estimated for the pre-treatment data, but the over-dispersion in the post-treatmentdata is assumed to be proportional to that of the pre-treatmentdata (the ratio used is based on published estimates of over-dispersion ratios in the host/parasite species you have selected)
- This method may give misleading results with fewer than five observations due to unstable estimates of over-dispersion
- This is the preferred method when the sample size is greater than or equal to 5, and where fewer than three post-treatment observations are non-zero

Results from the BNB method (Denwood et al.) version C

Classification: Susceptible

Test for Resistance: p = 0.198; Test for Susceptibility: p = 0.013

Notes:

- This method is parametric, and assumes that the data follow a negative binomial distribution: the classification will be unavailable if the multiplication factor you entered does not match the data
- The over-dispersion is not estimated from the data, but is assumed to follow published estimates for typical over-dispersion in the host/parasite species you have selected
- This method may give misleading results in some groups of animals where the population over-dispersion is in fact different to published estimates

- This is the only preferred method when the sample size is less than 5 (and is the only viable method with a sample size of 1)
